# In Memoriam—James Kitchen, M. D.

**Published:** 1894-11

**Authors:** 


### IN MEMORIAM—JAMES KITCHEN, M. D.,

### Consulting Physician of the Children's Homœopathic Hospital of Philadelphia.

*To the Board of Directors of the Children's Homœopathic Hospital of Philadelphia:*

Gentlemen :—The Committee appointed to prepare a minute of the late James Kitchen, M. D., who has been one of the consulting physicians of this Institution since its incorporation, respectfully submit the following record to be entered among our transactions :

James Kitchen, M. D., was born March 8th, 1800, in Philadelphia, and died August 19th, 1894.

He took an active interest in general professional matters pertaining to the homœopathic school of practice, and was in his earlier day a very skillful obstetrician.

His parentage was of Welsh origin, his father having been a merchant here in 1790.

He received his early literary education at the University of Pennsylvania, receiving the degree of Bachelor of Arts in 1819, subsequently entering the Medical Department as a student under Prof. Thomas A. Hewson and graduating in 1822.

He spent two years abroad in pursuit of additional medical knowledge, listening to lectures and receiving hospital instructions by the eminent teachers of that day.

In 1824 he settled in Spruce Street, near Second, and began his life work of practice.

At first he did not receive much of an income and became so much discouraged that he prepared to move to New Orleans, as a good opening seemed to offer in that city.

His father being seized with a sudden attack of illness, from which he died in a short time, he unpacked and settled in his father's house, his father having desired him to remain in Philadelphia and care for his mother and sisters.

He not only became prominent in his profession and a shining light, but had a very large practice, both general and obstetrical.

He served as Port Physician from 1832 to 1836, having been appointed to the Lazaretto, or quarantine station, in 1831.

Up to this time he was an "old-school" physician, but in 1839, after fifteen years of that mode of practice, he became a homœopath.

His old friend, Dr. Wm. S. Helmuth, had at the same time joined this school, together with Drs. Charles Neidhard, Jacob Jeanes, Walter Williamson, Samuel Freedley, David James, Isaac James, and others, almost all of whom were interested in the founding of the first homœopathic medical college in the world.

Although at first opposing the proposition, he at last consented to work with his brethren, and is found one of the original corporators in March, 1848. In October of the same year he drafted the diploma for the Homœopathic Medical College of Pennsylvania, and in 1862 he was made Corresponding Secretary of the Board of Managers.

At one time there was a homœopathic hospital organized, but owing to the lack of funds it never became a success, although he was appointed a professor of Clinical Medicine to it in 1852. It went down at the end of the first year.

In the same year he, with Dr. Wm. S. Helmuth, was made editor of the *Philadelphia Journal of Homœopathy*, which continued its career about four years. He was quite a writer and composed many articles on medical topics.

In 1853 he removed to the commodious house at 715 Spruce Street, which was his home during the remainder of his life.

For seventy years he was a practitioner of medicine, and we remember him as one of the most genial of men, kind and hospitable in character, a true friend, generous to a fault, and unobtrusive in his demeanor. In conversation he exhibited a good education and a high degree of intelligence and judgment.

He was a most exemplary man throughout his life, and this Institution can but feel that it has lost, not only one of its best friends, but one of its oldest and most honored supporters, and his memory will go down with links of friendship, brotherly love, and true devotion to the cause which he espoused in his earlier days.

Very respectfully submitted,

Bushrod W. James,
Napoleon B. Kelly,
*Committee.*

